# DISE, an ancient anti-cancer mechanism that senses mutational load in cancerous cells?

**DOI:** 10.18632/oncotarget.28466

**Published:** 2023-09-25

**Authors:** Monal Patel, Marcus E. Peter

**Keywords:** cancer, cell death, evolution, RNAi, short RNAs

Despite the multiple advances in therapy, cancer remains one of the most common causes of death globally. It is a systemic disease affecting people of all ages, originates at the level of single cells, which upon acquisition of mutations become neo-plastically transformed. Cell division is the biggest risk factor for accumulation of mutations [1], explaining why all multicellular organisms which evolved about 2 billion years ago, are prone to cancer. Given the recent achievements in cancer treatment with immune checkpoint blockade therapies, one could argue that multicellular organisms developed the immune system as a mechanism to eradicate cancerous cells [2]. However, the immune system arose relatively recent, ~500 million years ago [3]. Moreover, studies have shown that cancer cells can become resistant to the anticancer activity of both the innate and the adaptive immune system [4, 5]. Therefore, the immune system while important, is likely not the most vital machinery that emerged in multicellular organisms to prevent cancer formation; we believe that there must be other more effective and archaic anti-cancer mechanisms that are conserved during evolution. Of note, RNA interference (RNAi) is a highly conserved biological mechanism for silencing gene expression. While RNAi likely emerged as a defense tool against viruses and other foreign nucleic acids, it has also evolved to have other activities in the cells [6].

Our research has identified a new evolutionarily conserved RNAi-based form of cell death that targets essential survival genes, Death Induced by Survival gene Elimination (DISE) [7, 8]. DISE is effective against all cancers we tested. DISE was discovered through our work on CD95 and its ligand, CD95L, where we found that more than 80% of 26 different short interfering RNAs (siRNAs) and short hairpin RNAs (shRNAs) derived from the two genes, killed multiple cancer cell lines via simultaneous activation of multiple cell death pathways; and we were unable to find a way to inhibit this form of cell death [9]. We subsequently reported that CD95L is enriched in sequences that when converted to sRNAs are toxic to cells. In fact, we found that CD95L is processed into short RNAs (sRNAs) that are loaded into the RNA induced silencing complex (RISC) and kill cells through DISE [10, 11]. Later, we determined that all these toxic sRNAs did not kill cells by acting like typical siRNAs i.e., by silencing genes through complete complementarity to a section of the mRNA, but by working in a microRNA (miRNA)-like fashion i.e. using just a seed sequence and predominantly targeting the 3′UTR [12]. In fact, the shortest seed of 6 nucleotides was sufficient to have toxicity. The most toxic 6mer seeds were then identified in large arrayed screens testing all possible 4096 6mer seeds embedded in a 19 double stranded short RNA (sRNA) in three human and three mouse cancer cell lines [12, 13]. We assessed the toxicity of positions 2–7 in the guide strand of the sRNA by blocking the loading of the passenger strand via two 2′-O-methylation of its positions 1 and 2 [14]. We found that the most toxic seeds were all G-rich and they targeted C-rich sequences in the 3′UTRs of essential survival genes. The rules of “6mer seed toxicity” were universal and independent of cancer type or species. When put into the context of the coevolution of the ~2300 known human miRNAs with the 3′UTRs of genes, especially survival genes [12, 15], it became clear that we discovered a death mechanism based on miRNAs selectively targeting survival genes. Consequently, we found that some of the seeds of major tumor suppressive and death inducing miRNAs such as miR-34a-5p or miR-15/16-5p have G rich 6mer seeds and kill cells by targeting survival genes [12, 16]. More support for DISE as an evolutionary conserved mechanism came with the discovery that certain viral miRNAs can be toxic to cells through this mechanism [16], and also for HIV-1, we observed a contribution of DISE to its cytopathicity [17]. Based on the evolutionary conservation of miRNAs we estimated this mechanism to be >800 million years old [12].

We hypothesize that DISE is part of a natural anti-cancer mechanism that is triggered when cells accumulate cancer causing mutations. So, if DISE is activated when cells sense a high mutational load, then most chemotherapeutic drugs should also trigger the DISE mechanism as they are genotoxic and acutely induce mutations. Indeed, our data show that different chemotherapeutic reagents resulted in induction of RISC-bound sRNAs and miRNAs that carry toxic 6mer seeds, suggesting that these drugs kill cancer cells, at least in part, by triggering DISE [12]. We subsequently focused on ovarian cancer, a leading cause of cancer death in women. High-grade serous ovarian cancer has a dismal prognosis, with 65% of patients dying within 5 years following diagnosis. Current treatment strategies including debulking surgery and combination drug treatment with the platinum-based drug carboplatin and paclitaxel as first-line treatment and the addition of angiogenesis and PARP inhibitors to treat Stage III/IV and platinum-resistant cancer, have extended the survival of women diagnosed with ovarian cancer, but they have not been curative. Eventually, platinum resistance renders repeated courses of treatment with platinum-based drugs ineffective, resulting in a 9- to 12-month survival window for these patients. We performed a comprehensive literature search and found that a huge number of different miRNAs have been linked to platinum resistance in ovarian cancer [18]. We provided an explanation for these observations by showing that it may not be the nature of the individual miRNAs but the balance of toxic and nontoxic 6mer seed containing miRNAs in the RISC that affects treatment outcome in ovarian cancer. Interestingly, resistance to platinum treatments rendered ovarian cancer cells more sensitive to the effects of our toxic sRNAs *in vitro*, as well as in mice and in ovarian cancer patients [18]. We hypothesized that in normal cells, naturally occurring miRNAs out-compete toxic sRNAs from loading into the RISC. However, in cancer cells, miRNA expression is globally downregulated [19, 20], allowing therapeutic sRNAs to promote cancer cell death. Consequently, in preclinical studies of xenografted human ovarian cancer in mice, treatment with toxic seed containing sRNAs coupled to HDL mimetic nanoparticles slowed down tumor growth without any signs of toxicity in the treated mice [21].

**Figure 1:**
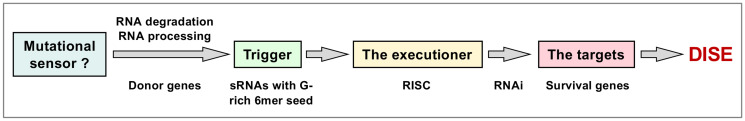
Schematic of the postulated DISE mechanism. When the mutational load in a precancerous cell rises above a certain level, an as yet unknown sensor mechanism is activated resulting in the generation of short RNA fragments or mature miRNAs that carry toxic 6mer seeds. These sRNAs get loaded into the RISC and target complementary C-rich seed matches located in the mRNAs of a number of genes critical for cell survival. The resulting combination of cell death pathways that get activated is referred to as death induced by survival gene elimination, DISE.

Our data are consistent with DISE being part of an ancient anti-cancer mechanism that could be used to treat cancer. The DISE mechanism therefore qualifies to be the ancient anti-cancer mechanism we postulated above. The fact that chemotherapy has features of DISE - both induce DNA damage [9, 22], is consistent with DISE being a mechanism that is triggered by an increased mutational load. We now have the downstream components of the DISE mechanism: (1) The trigger - sRNAs with toxic G-rich seeds; (2) The machinery that executes DISE - the RISC; and (3) The ubiquitous targets - essential survival gene (Figure 1). What is missing at this point is the nature of the upstream sensor that senses whether the mutational load of a cell is raised above the level of what a cell can tolerate and what is required for evolution to occur. This sensor may involve known signaling components that sense DNA stress (e.g., ATM/ATR), but could also be entirely unknown. Another question is how cells with high levels of DNA strand breaks, including T and B cells with rearranging B cell and T cell receptor genes, prevent activation of DISE.

The discovery of DISE as a new anti-cancer mechanism holds great promise to eventually develop a pan cancer therapy. In order to convert this into an effective and safe form of therapy, however, several questions need to be addressed, most importantly whether conditions exist under which triggering of DISE affects normal cells.
